# LONG-TERM EFFECTS OF SPINAL CORD STIMULATION COMBINED WITH A PHYSIOTHERAPIST-LED EXERCISE PROGRAMME AND PHYSICAL ACTIVITY ON PRESCRIPTION FOR CHRONIC NEUROPATHIC PAIN

**DOI:** 10.2340/jrm.v58.46459

**Published:** 2026-10-14

**Authors:** Raquel GOTTFRIDSSON, Emma VARKEY, Kliment GATZINSKY, Helena THÖRN, Mats BÖRJESSON, Daniel ARVIDSSON, Sven-Egron THÖRN, Axel WOLF, Paulin ANDRÉLL

**Affiliations:** 1 Department of Anaesthesiology, Intensive Care Medicine and Pain Medicine, Sahlgrenska University Hospital/Östra, Gothenburg, Region Västra Götaland, Sweden; 2 Department of Anaesthesiology and Intensive Care, Institute of Clinical Sciences, Sahlgrenska Academy, University of Gothenburg, Gothenburg, Sweden; 3 Department of Occupational Therapy and Physical Therapy, Sahlgrenska University Hospital/Östra, Gothenburg, Region Västra Götaland, Sweden; 4 Department of Health and Rehabilitation/Physiotherapy, Institute of Neuroscience and Physiology, Sahlgrenska Academy, University of Gothenburg, Gothenburg, Sweden; 5 Department of Neurosurgery, Sahlgrenska University Hospital, Gothenburg, Region Västra Götaland, Sweden; 6 Department of Clinical Neuroscience, Institute of Neuroscience and Physiology, Sahlgrenska Academy, University of Gothenburg, Gothenburg, Sweden; 7 Department of Oncology and Palliative Medicine, Halland Regional Hospital, Varberg, Sweden; 8 Department of Medicine, Geriatrics and Emergency Care, Centre for Lifestyle Intervention, Sahlgrenska University Hospital, Gothenburg, Region Västra Götaland, Sweden; 9 Department of Molecular and Clinical Medicine, Institute of Medicine, Sahlgrenska Academy, University of Gothenburg, Gothenburg, Sweden; 10 Department of Food and Nutrition and Sport Science, Faculty of Education, University of Gothenburg, Gothenburg, Sweden; 11 Institute of Health and Care Sciences, Sahlgrenska Academy, University of Gothenburg, Gothenburg, Sweden; 12 Centre for Person-Centred Care, Sahlgrenska Academy, University of Gothenburg, Gothenburg, Sweden; 13 Institute of Nursing and Health Promotion, Oslo Metropolitan University, Oslo, Norway

**Keywords:** chronic pain, neuropathic pain, physical activity, physical therapy modalities, spinal cord stimulation

## Abstract

**Objective:**

Assess the effects of spinal cord stimulation and add-on physical activity interventions on pain, physical activity levels, and health-related quality of life.

**Design:**

Prospective single-arm.

**Subjects/Patients:**

35 adults who underwent spinal cord stimulation device implantation for neuropathic pain completed the study.

**Methods:**

Three months after implantation, participants initiated a 6-month individualized physiotherapist-led exercise programme, followed by 12 months of physical activity on prescription. Neuropathic pain intensity (Numeric Rating Scale, 0–10), physical activity (accelerometer), and health-related quality of life (EuroQol 5-dimensions, visual analogue scale, EQ-5D VAS; 0–100) were assessed at baseline and 3, 9, and 21 months after implantation.

**Results:**

Neuropathic pain was reduced from baseline at 9 and 21 months (mean [SD] = 6.6 [1.7] at baseline; 4.8 [2.3] at 9 months, *p* = 0.003; 4.8 [2.1] at 21 months, *p* = 0.003). EQ-5D VAS scores increased significantly by 16 (*p* = 0.027) and 15 (*p* = 0.003) points at 9 and 21 months, respectively. No changes in physical activity were observed. Changes from 3 months were not statistically significant at 9 or 21 months for any outcomes.

**Conclusion:**

The initial effects of spinal cord stimulation on pain and health-related quality of life remained after the introduction of physical activity interventions, with no additional improvements observed beyond 3 months after implantation.

## LAY ABSTRACT

Chronic nerve pain is an intense type of pain that is difficult to treat with medications alone. Spinal cord stimulation and rehabilitation (including physiotherapy) are alternative ways of managing chronic pain when medications do not provide sufficient pain relief. We evaluated the long-term effects of spinal cord stimulation combined with physical activity interventions on pain intensity, physical activity levels, and quality of life. A total of 35 participants with chronic nerve pain completed the study. Three months after spinal cord stimulation device implantation, participants started a 6-month physiotherapist-led exercise programme. After completing the programme, they were prescribed individualized physical activity for 12 months. Participants experienced pain relief and improved quality of life at the 9- and 21-month follow-ups. The effects were sustained, but no further improvements were seen beyond 3 months after implantation. Physical activity levels were very low among participants and did not increase over the follow-up period.

Chronic neuropathic pain is a severe pain condition caused by a lesion or disease of the somatosensory system, which leads to disability and impairs health-related quality of life (HRQL) and physical functioning (1). Pharmacological treatment is challenging (2). Techniques such as spinal cord stimulation (SCS) have been developed to aid the treatment of resistant neuropathic pain (2, 3). SCS has been shown to reduce pain intensity and improve HRQL, physical functioning, and mobility; however, its long-term treatment effects are variable (4–6). Rehabilitation and physiotherapeutic interventions, including physical activity (PA), commonly applied to chronic nociceptive and nociplastic pain conditions, have also been proposed for the treatment of neuropathic pain (7–9). PA is traditionally defined as any bodily movement that results in increased energy expenditure (10). Ample evidence supports the positive effects of PA on health and longevity, and regular PA of sufficient intensity and duration is considered essential for the prevention and treatment of several diseases (11). PA is associated with beneficial effects among patients with chronic pain, including pain relief, increased HRQL, and reduced risk of inactivity-related diseases (e.g., cardiovascular disease, type 2 diabetes, obesity) (11). However, individuals with chronic pain often exhibit low PA levels and do not meet the recommendations of the World Health Organization (WHO) for 150–300 min/week of moderate-to-vigorous physical activity (MVPA) (11–13). Low PA levels and other lifestyle factors may underlie the association between chronic pain and cardiovascular disease (14). While PA interventions may reduce neuropathic pain intensity and mitigate its unpleasantness, there is limited evidence regarding physiological effects and the mechanistic interaction between PA and neuropathic pain (15).

A recent review and meta-analysis indicated that the effects of rehabilitation and PA interventions in patients with chronic neuropathic pain may be comparable to those observed in the general chronic pain population (7). However, long-term adherence to PA recommendations following physiotherapeutic and rehabilitation interventions remains a challenge. Physical activity on prescription (PAP) has been developed and implemented in Sweden to facilitate long-term adherence to PA in chronic conditions, including chronic pain (16). Tailored PAP includes a written prescription and regular follow-ups to maintain motivation to perform PA and increase PA levels (16, 17). PAP has been shown to effectively increase PA levels, contribute to increased HRQL, and improve metabolic risk profile (18).

Currently, there is a lack of studies assessing the effects of SCS in relation to rehabilitation outcomes and in combination with PA interventions. We previously published findings from a prospective single-arm clinical study indicating improvements in pain, HRQL, and self-efficacy 3 months after SCS device implantation in a clinical sample of patients with chronic neuropathic pain (19). Participants subsequently completed a 6-month physiotherapist-led exercise programme and, thereafter, 12 months of PAP. The present study is a long-term follow-up aimed to assess treatment effects on self-reported pain intensity, PA levels, and HRQL at 9 and 21 months after SCS device implantation. We hypothesized that SCS would facilitate PA, increasing MVPA levels, and that the PA interventions would complement the beneficial effects of SCS on pain relief.

## METHODS

### Ethical approval

The study was conducted in accordance with the Declaration of Helsinki (revised in 2013) and was approved by the Gothenburg Central Ethical Review Board and the Swedish Ethical Review Authority (registration number 450-16, supplementary applications registration numbers T749-18; 2019-05318, 2023-03398-02). All participants received oral and written information and those who subsequently gave both oral and written informed consent were enrolled in the study. Withdrawal was permitted without the need for further explanation.

### Study design

The 3-phase complex intervention study examined treatment with SCS up to 3 months after implantation (Phase 1), a 6-month add-on physiotherapist-led exercise programme (Phase 2), and 12 months of PAP for chronic neuropathic pain (Phase 3) (Fig. 1). The study design and methods have been described previously (19). The last 2 phases of the study (2 and 3) are presented in detail in the present paper. The study has been registered in ClinicalTrials.gov (NCT03740763) and in the Västra Götaland Region registry (https://www.researchweb.org/is/vgr/project/216271).

**Fig. 1:**
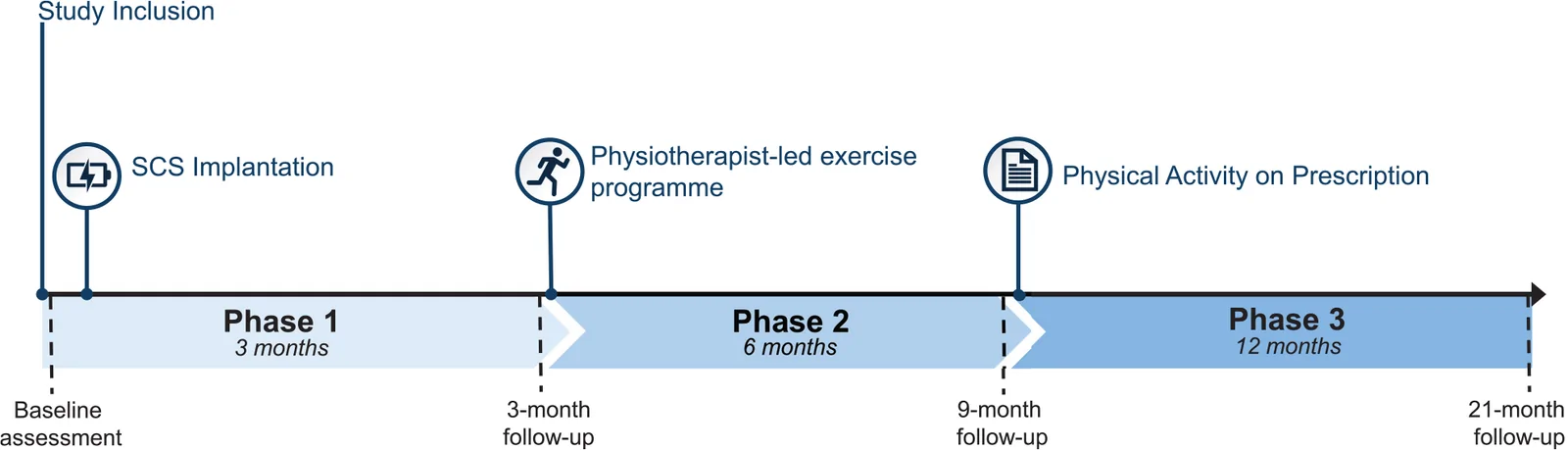
Study design. SCS: spinal cord stimulation.

### Participants

Adults (> 18 years) with chronic neuropathic pain with pain intensity ≥ 5 on the Numeric Rating Scale for Pain (NRS; 0–10 point scale, 0 = no pain, 10 = worst imaginable pain) and pain duration for ≥ 6 months, who were accepted for SCS treatment at the Pain Centre or Neurosurgical Department at the Sahlgrenska University Hospital, Gothenburg, Sweden, were assessed for study eligibility according to the inclusion and exclusion criteria presented in Table I (19).

**Table I:** Inclusion and exclusion criteria for the complex intervention study

Inclusion criteria Neuropathic pain ≥ 6 months Pain intensity ≥ 5 on the NRS Neuropathic pain with neuroanatomical correlation SCS aimed to cover ≥ 50% of the painful area Physical and psychological function permitting SCS treatment and execution of physiotherapist-led exercise
Exclusion criteria Other disabling pain condition Serious untreated psychiatric conditions Alcohol and substance abuse Pregnancy Poor adherence (e.g., cognitive dysfunction) Malignancy with short life expectancy Insufficient knowledge of the Swedish language

NRS: Numeric Rating Scale for pain (scale 0–10; 0 = no pain, 10 = worst imaginable pain); SCS: spinal cord stimulation.

### Procedure

Patients who met the inclusion criteria and accepted participation in the study were implanted with an SCS device (Abbott Laboratories, Chicago, IL, USA; Boston Scientific, Marlborough, MA, USA; Medtronic, Minneapolis, MN, USA; and Nevro Corp, Redwood City, CA, USA within routine clinical practice) after a successful 2-week test trial and proceeded with Phase 1 (19). After implantation, participants were randomized 1:1 to either Active SCS group or Inactive SCS group. For those randomized into the Inactive SCS group, devices were switched off for the first 6 months, whereas devices remained active for those in the Active SCS group (19). Participants in the Inactive SCS group who requested activation of the SCS device were permitted crossover to the Active SCS group. When Phase 1 was completed, all participants moved to Phase 2, a physiotherapist-led exercise programme, and subsequently to Phase 3, PAP (see Fig. 1).

### Phase 2: Physiotherapist-led exercise programme

Three months after the implantation of an SCS device, participants started an individually tailored 6-month exercise programme led by physiotherapists specialized in pain and pain rehabilitation. The programme was based on muscular strength, endurance, and core stability, with additional aerobic exercises individually tailored according to clinical assessment and the patient’s preferences and needs (e.g., age, relevant diagnoses, mobility status, and preference for gym-based or home-based exercise). The recommendation was to perform exercise sessions of approximately 60 min twice weekly, at the Pain Centre in Gothenburg, at a local gym, or at a local physiotherapy clinic, depending on the participant’s preference. Home-based exercise was also possible, and, for some participants, gradual progression starting with shorter and more frequent sessions was necessary.

### Phase 3: Physical activity on prescription

After completing the 6-month physiotherapist-led exercise programme, participants were offered PAP to continue exercising on their own. PAP entails the following individualized steps: (*i*) person-centred dialogue regarding current PA levels, motivations, and preferences; (*ii*) the recommended PA dosage; (*iii*) adjustment of ideal PA dosage according to concomitant diseases and disabilities; and (*iv*) regular follow-ups (16). PAP was prescribed by the study physician (PA), in consultation with the treating physiotherapist at the Pain Centre. Prescriptions comprised both strength and aerobic exercises and were individualized based on clinical assessments by the study physician and physiotherapist, the participant’s pain locations, underlying diseases (including other pain conditions), and the participant’s own preferences. The prescription included the duration, frequency, and intensity of PA according to the Frequency, Intensity, Time, Type of PA (FITT) principle (20). Follow-ups were conducted by telephone by a research nurse 12 and 15 months after SCS device implantation to follow up PAP and the need for prescription renewal. In cases where concerns were brought up by participants, the study physician was contacted. Follow-ups at 12 months after the first PAP prescription, i.e., 21 months after SCS device implantation, was performed by the study physician. PAP was renewed by the study physician by the end of the study or more frequently, if requested by the participant. A plan for continued follow-up of PAP within primary care was established at the last study visit.

### Outcome measures

Patient-reported outcome measures (PROMs) and other outcome measures have been described in detail in the Phase 1 report (19). No additional outcome measures were employed for Phases 2 and 3. Outcome assessments were conducted at baseline and the 3-, 9-, 12-, 15-, and 21-month follow-ups after permanent SCS device implantation, either on site or digitally depending on participant circumstances and COVID-19 restrictions. Intermediate assessments at 12 and 15 months were performed by phone to facilitate long-term follow-up and PAP. PROM questionnaires were sent by post to each participant, although some participants completed the questionnaires during study visits. In cases where participants did not respond within 2 weeks, research nurses reached out by phone and could resend questionnaires if needed.

*Pain.* Pain outcomes were self-reported and assessed using the NRS. Neuropathic pain intensity was reported at rest and during movement on the previous day and over the previous week. Average neuropathic pain intensity was calculated for each participant based on the previous week’s values. Total pain assessments consisted of both neuropathic pain and pain of other aetiologies reported at rest and during movement over the previous week. The minimal clinically important difference (MCID) for the NRS has traditionally been estimated at 2 points for chronic pain (21). A 1–3 points change on the NRS has been suggested to be clinically significant for patients treated with SCS (22).

*Physical activity measurements.* Objectively assessed PA data were collected at baseline and at 3, 9, and 21 months after SCS device implantation using an Axivity AX3 accelerometer (Axivity Ltd, Newcastle upon Tyne, UK). Participants wore the accelerometer for 24 h a day for 7 consecutive days at each assessment time point, except during contact with water. The accelerometer was placed in an elastic belt around the waist and positioned over the right hip to ensure that bodily movement and PA intensity were recorded accurately. Collection and conversion of raw data have previously been described (19).

PA measurements were expressed in relative and absolute terms (23, 24). Absolute PA intensity is usually expressed as metabolic equivalents of task (METs), where 1 MET is the oxygen consumption during rest (3.5 ml/min/kg). Its measures include energy expenditure, locomotion speed, and mechanical work, all of which represent the same absolute intensity regardless of who is performing the activity. Relative PA intensity refers to absolute intensity in relation to individual maximal PA capacity (determined by individual maximal oxygen consumption; VO2max) and differs depending on individual cardiorespiratory fitness (24). Relative PA intensity can be determined as the proportion of estimated VO2max during activity, defined as moderate (> 46%), vigorous (> 64%), and very vigorous (> 91%). The VO2max for the participants included in this study was set to 32 ml/min/kg according to previous data measured in a representative sample of patients at the Pain Centre. Sedentary time (SED) was expressed in absolute terms (METs < 1.5), as there are no existing references values for relative SED (24, 25), and MVPA was expressed as ≥ 46% VO2max.

In addition, self-reported PA was assessed using the Saltin–Grimby Physical Activity Level Scale (SG-PALS), which has a score range from 1–4, where higher scores indicate higher PA levels (26).

*Additional PROMs.* All PROMs used in the study have been described in detail previously (19). The visual analogue scale (VAS) of the European Quality of Life instrument (EuroQoL 5-dimensions questionnaire; EQ-5D VAS; a 0–100 point scale; where 0 = worst health, 100 = best possible health) was used to assess HRQL (27). The MCID for the EQ-5D VAS is usually set to > 7 or > 10 points (28). HRQL was also assessed using the RAND-36 questionnaire (8 domains, score range 0–100 each; higher scores indicate better HRQL). Self-efficacy was assessed using the Arthritis Self-Efficacy Scale (ASES; score range 0–100; higher scores imply higher self-efficacy) and the Self-Efficacy for Exercise Scale (SEE; score range 0–90; higher scores imply higher self-efficacy). Fear of movement (i.e., kinesiophobia) was assessed using the TAMPA scale of Kinesiophobia (score range 17–68; higher scores imply more kinesiophobia; cut-off of 37 indicating high levels of kinesiophobia).

*Patient satisfaction.* Patient satisfaction was self-reported using an NRS scale (0–10-point scale; 0 = not satisfied, 10 = very satisfied) at 9 and 21 months after SCS device implantation (29).

### Adverse events

Adverse events (AEs) were self-reported throughout the study and specifically addressed during the study visits. Classification was made in accordance with the Department of Health and Human Services’ Common Terminology Criteria for Adverse Events (CTCAE) version 5.0 (grade score 1–5; grades 3–5 imply serious AEs) (30).

### Statistics

Sample size calculations have been reported previously (19). IBM SPSS Statistics version 29 (IBM Corp, Armonk, NY, USA) was used for statistical analyses. Questionnaires were securely stored anonymously. Data distribution was assessed for all variables before the analyses through visual inspection of histograms (31). Variables with approximately symmetric distribution were presented as means with standard deviations (SD) and were analysed with the paired *t*-test. Variables with skewed distribution, extreme values, or several missing values were presented as medians with interquartile ranges and ranges (Q1–Q3; min–max) and were analysed with the Wilcoxon signed rank test. McNemar’s test was used to compare the proportions of participants who reported ≥ 50% pain relief across the follow-ups. Statistical significance was reached when *p* < 0.05; all *p*-values were two-sided and corrected for multiple analyses using the Bonferroni correction regarding the number of analyses made for each variable. In cases where the corrected *p*-value exceeded 1, it was approximated to 1.0. If questionnaire values were missing at the 21-month follow-up, values were imputed from the 12- or 15-month follow-up. The primary outcome of interest of the complex intervention study was patients reporting ≥ 50% pain relief at 3 months, which has been reported previously in a separate publication (19).

## RESULTS

### Participants

A total of 48 participants were included between May 2018 and October 2021; 42 participants were implanted with the SCS device and randomized to Active SCS (*n* = 21), or Inactive SCS (*n* = 21) (19). A total of 19 participants in the Inactive SCS group requested activation of the device due to considerable pain reduction during test trial, and they crossed over to the Active SCS group. A total of 35 participants completed the 9- and 21-month follow-ups (Fig. 2). Seven participants left the study between randomization and the 9-month follow-up. No further withdrawals occurred. The study ended with the last study visit in November 2023. A detailed flowchart from screening to the 3-month follow-up has been published previously (19). Demographic characteristics of the study population are presented in Table II.

**Fig. 2:**
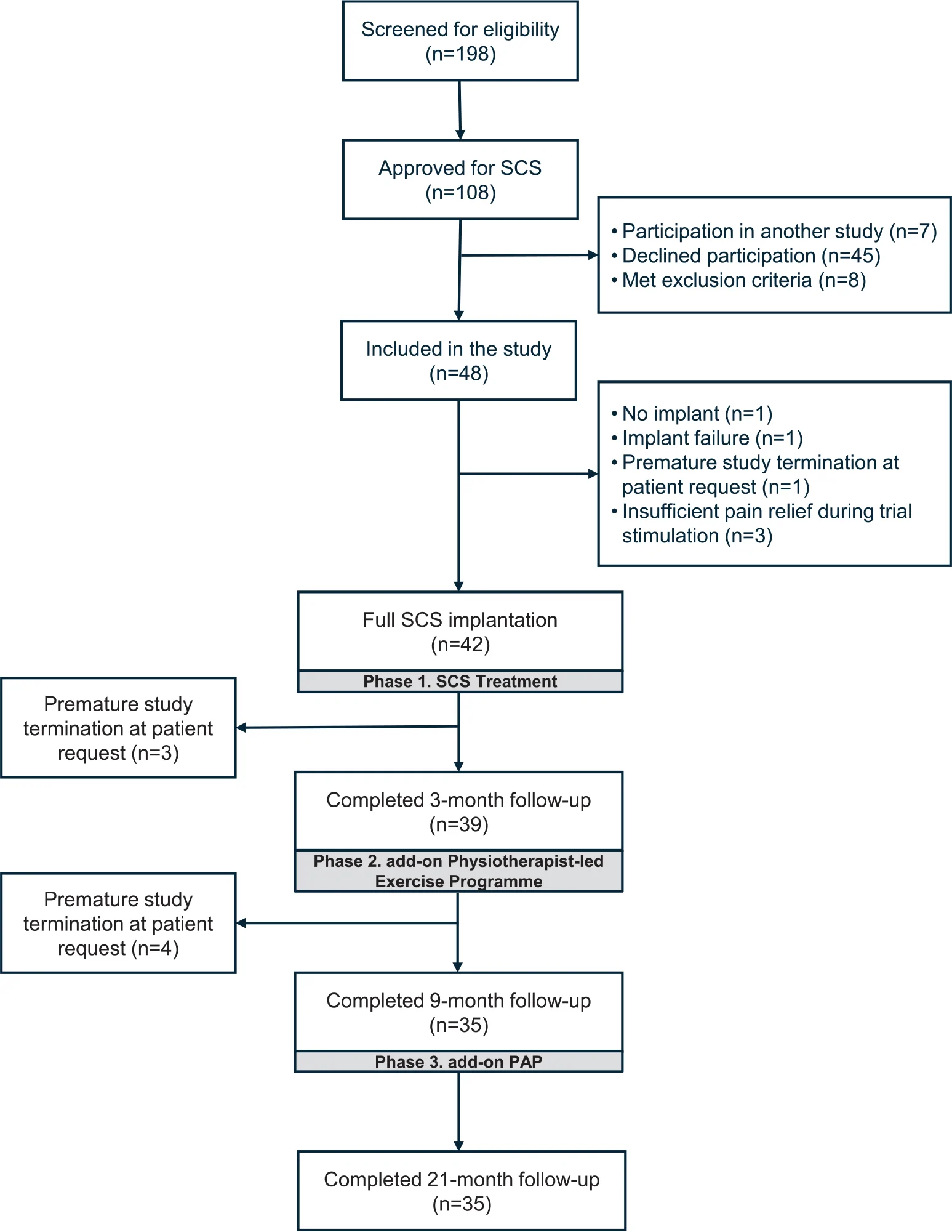
Flowchart. SCS: spinal cord stimulation; PAP: physical activity on prescription.

**Table II:** Baseline patient characteristics for all included patients and those completing the 21-month follow-up visit

Item	All included patients (*n* = 48)	Patients completing the 21-months’ follow-up (*n* = 35)
Age, years, mean (SD)	53 (13)	53 (13)
Median (min; max)	54 (29; 77)	53 (29; 77)
Gender, women/men, *n* (%)	27/21 (56/44)	20/15 (57/43)
Pain duration, years, *n* (%)		
< 1 year	0 (0)	0 (0)
1–5 years	27 (56)	22 (63)
6–10 years	12 (25)	9 (26)
>10 years	9 (19)	4 (11)
Education, *n* (%)		
Elementary school	7 (15)	6 (17)
High school	17 (35)	10 (29)
College/University	13 (27)	10 (29)
Other	10 (21)	8 (23)
Missing	1 (2)	1 (2)
Occupational status, *n* (%)		
Employment/Studies 100%	7 (15)	3 (9)
Employment/Studies 75%	4 (8)	4 (11)
Employment/Studies 50%	5 (10)	4 (11)
Employment/Studies 25%	2 (4)	2 (6)
Job applicant	2 (4)	2 (6)
Sick leave 100%	18 (38)	14 (40)
Sick leave 75%	0 (0)	0 (0)
Sick leave 50%	0 (0)	0 (0)
Retired	9 (19)	6 (17)
No employment/studies/sick leave	1 (2)	0 (0)
Marital status, *n* (%)		
Married/Cohabiting	37 (77)	26 (74)
Single	11 (23)	9 (26)
Smoking status, *n* (%)		
Active/Periodic smoker	15 (31)	7 (20)
Former smoker	20 (42)	17 (49)
Nonsmoker	13 (27)	11 (31)
Snuff, *n* (%)		
Active/Periodic	16 (33)	9 (25)
Former	5 (11)	3 (9)
Never	23 (48)	20 (57)
Missing	4 (8)	3 (9)
BMI, kg/m^2^, mean (SD)	28 (4)	28 (4)
Median (min; max)	28 (20; 37)	28 (20; 37)
Neuropathic pain location, *n* (%)		
Upper extremity	5 (10)	4 (11)
Lower extremity	41 (85)	29 (83)
Trunk	8 (17)	5 (14)
Indication for SCS treatment, *n* (%)		
CRPS	11 (23)	6 (17)
PSPS II	31 (65)	23 (66)
Other	6 (12)	6 (17)
Pain in other locations, *n* (%)	41 (85)	31 (89)
Pain medication, *n* (%)		
Paracetamol	26 (54)	17 (49)
Anti-inflammatory drugs	12 (25)	9 (26)
Acetylsalicylic acid with caffeine	1 (2)	1 (3)
Opioids	31 (65)	25 (71)
Antidepressants	24 (50)	17 (49)
Anticonvulsants	22 (46)	18 (51)
Sedatives and Z-drugs	9 (19)	6 (17)

Percentages are based on available data. Other indications for SCS treatment include chronic persistent post-surgical pain of different causes, post-traumatic neuropathic pain, or chemotherapy-induced neuropathic pain. Anti-inflammatory drugs include nonsteroidal anti-inflammatory drugs (NSAIDs) and Selective COX-2 inhibitors. Z-drugs: nonbenzodiazepines, i.e., zopiclone, zolpidem.

SD: standard deviation; SCS: spinal cord stimulation; CRPS: complex regional pain syndrome; PSPS II: persistent spinal pain syndrome.

### Outcome measures

Data from the 3-month follow-up have been previously published but are included herein for reference (19).

*Pain.* Pain intensity outcomes are presented in Table III. The proportion of patients reporting ≥ 50% pain relief increased from 3 (11.5%) at 3 months to 7 (25%) at 9 months, and remained stable through to 21 months (*n* = 7, 21%). However, these changes were not statistically significant after Bonferroni correction at any follow-up. Statistically significant pain reduction from baseline was found regarding all neuropathic pain intensity outcomes and total pain during movement. The mean neuropathic pain average score decreased from 6.6 (SD = 1.7) at baseline for those with complete follow-up to 4.8 (SD = 2.3, *p* = 0.005) at 9 months, which was sustained to the 21-month follow-up (4.8, SD = 2.1, *p* = 0.005) (Table III). The median total pain in movement score was reduced from 7.0 (Q1–Q3 = 6–8; min–max = 2–10) to 6.0 (Q1–Q3 = 4–8; min–max = 0–10; *p* = 0.025) at 9 months and to 6.0 (Q1–Q3 = 5–8; min–max = 0–10; *p* = 0.03) at 21 months. Improvements in total pain at rest from baseline did not reach statistical significance after Bonferroni correction at 9 or 21 months (*p* = 0.11 and *p* = 0.095, respectively) (Table III). There were no statistically significant differences between follow-ups at 3, 9, and 21 months for any of the pain outcomes.

**Table III:** Primary and secondary outcomes: changes from baseline at the 3-, 9, and 21-month follow-ups

Factor	Baseline values										
	All patients (*n* = 48)	Complete follow-up (*n* = 35)	3 months (*n* = 26–28)	Mean difference from baseline [95% CI]	*p*-value	9 months (*n* = 27–30)	Mean difference from baseline [95% CI]	*p*-value	21 months (*n* = 31–33)	Mean difference from baseline [95% CI]	*p*-value
*Primary outcome*											
Pain relief ≥ 50%, *n* (%)			3 (11.5)			7 (25%)			7 (21%)		
*Secondary outcomes*											
Pain											
NRS (0–10)											
Pain relief ≥ 30%, *n* (%)			12 (46)			13 (46)			16 (49)		
Pain relief 0–29%, *n* (%)			12 (46)			10 (36)			12 (36)		
Increased pain, *n* (%)			2 (8)			5 (18)			5 (15)		
Neuropathic pain, average, mean (SD)	6.7 (1.7)	6.6 (1.7)	4.5 (1.6)	–1.6 [–2.2 to –1.1]	0.005*^a^	4.8 (2.3)	–1.6 [–2.5 to –0.7]	0.005*^a^	4.8 (2.1)	–1.7 [–2.5 to –1]	0.005*^a^
Neuropathic pain last week at rest, mean (SD)	6.2 (1.9)	6.0 (1.8)	3.9 (1.7)	–1.8 [–2.5 to –1.0]	0.005*^a^	4.5 (2.2)	–1.4 [–2.3 to –0.4]	0.035*^a^	4.5 (2.2)	–1.5 [–2.4 to –0.7]	0.005*^a^
Neuropathic pain last week during movement, mean (SD)	7.3 (1.9)	7.1 (1.9)	5.1 (2.0)	–1.5 [–2.1 to –1.0]	0.005*^a^	5.1 (2.4)	–1.9 [–2.8 to –1.0]	0.005*^a^	5.2 (2.3)	–1.9 [–2.7 to –1.2]	0.005*^a^
Total pain last week at rest, median (Q1–Q3; min–max)	6.0 (5–8; 3–10)	6.0 (5–8; 3–10)	5.0 (3–6; 1–9)		0.02*^b^	5.0 (3–7; 2–9)		0.11^b^	5.0 (6–8; 0–9)		0.095^b^
Total pain NRS last week during movement, median (Q1–Q3; min–max)	7.0 (6–8.3; 2–10)	7.0 (6–8; 2–10)	6.0 (5–8; 2–9)		0.03*^b^	6.0 (4–8; 0–10)		0.025*^b^	6.0 (5–8; 0–10)		0.03*^b^
Physical activity											
Accelerometer											
SED, min/day, median (Q1–Q3; min–max)	661 (596–722; 487–817)	663 (594–737; 487–817)	670 (635–726; 560–820)		0.66^b^	652 (596–727; 485–861)		1.0^b^	652 (601–727; 518–861)		1.0^b^
MVPA, min/week, median (Q1–Q3; min–max)	13 (6.7–42; 3.2–223)	15 (8.6–49; 3.6–223)	17 (4.9–65; 1.3–281)		1.0^b^	11 (5.6–39; 1.8–270)		1.0^b^	12 (5.2–57; 2.3–263)		1.0^b^
HRQL											
EQ-5D VAS (0–100), mean (SD)	39 (15)	40 (16)	60 (20)	14 [5–23]	0.015*^a^	56 (23)	14 [4–23]	0.045*^a^	55 (21)	14 [7–22]	0.005*^a^
RAND-36 (0–100)											
Physical Functioning, median (Q1–Q3; min–max)	50 (30–60; 5–90)	50 (40–63; 15–90)	63 (40–70; 15–90)		0.125^b^	60 (28–79; 5–100)		0.69^b^	55 (30–75; 5–100)		1.0^b^
Role Functioning/Physical, median (Q1–Q3; min–max)	0 (0–0; 0–75)	0 (0–0; 0–50)	25 (0–44; 0–100)		0.035*^b^	0 (0–50; 0–100)		0.135^b^	0 (0–25; 0–100)		0.095^b^
Pain, median (Q1–Q3; min–max)	23 (10–35; 0–48)	23 (10–34; 0–48)	45 (23–55; 0–70)		0.005*^b^	40 (23–45; 0–90)		0.02*^b^	33 (23–45; 0–90)		0.005*^b^
General health, median (Q1–Q3; min–max)	45 (35–55; 15–90)	45 (35–58; 25–90)	55 (45–70; 15–90)		1.0^b^	53 (40–70; 10–95)		0.99^b^	45 (33–45; 10–100)		1.0^b^
Energy/Fatigue, median (Q1–Q3; min–max)	25 (15–50; 0–85)	30 (15–52; 5.0–85)	55 (30–70; 5–80)		0.045*^b^	55 (35–65; 0–95)		0.29^b^	45 (35–60; 0–85)		0.21^b^
Social functioning, median (Q1–Q3; min–max)	38 (25–50; 0–100)	38 (19–50; 0–100)	75 (38–75; 25–100)		0.005*^b^	57 (38–75; 0–100)		0.07^b^	63 (32–60; 13–100)		0.005*^b^
Role Functioning/Emotional, median (Q1–Q3; min–max)	67 (0–100; 0–100)	67 (0–100; 0–100)	100 (42–100; 0–100)		0.75^b^	100 (8–100; 0–100)		1.0^b^	100 (33–100; 0–100)		0.15^b^
Emotional well-being, median (Q1–Q3; min–max)	60 (44–80; 12–96)	64 (48–80; 12–96)	72 (48–88; 16–96)		1.0^b^	72 (56–92; 20–100)		1.0^b^	72 (50–86; 4–100)		1.0^b^
Patient satisfaction											
NRS (0–10)											
Satisfaction with pain relief, median (Q1–Q3; min–max)			7.0 (5–8.8; 0–10)			7.0 (3.3–8.8; 0–10)			8.0 (4.0–8.5; 0–10)		
Treatment acceptability, median (Q1–Q3; min–max)			8.0 (6.3–9.8; 1–10)			7.5 (4.3–10; 2–10)			8.0 (5.0–9.0; 0–10)		
Health care, median (Q1–Q3; min–max)			9.0 (8–10; 2–10)			9.0 (5.3–10; 2–10)			8.0 (6–10; 0–10)		

Analyses are relative to baseline values, and percentages are based on available data.

* *p*-value significant when <0.05. *p*-values are Bonferroni-corrected for multiple comparisons throughout all tests.

a Symmetrical distribution, mean (SD), and *t*-test for mean comparison.

b Not symmetrical distribution, median (Q1–Q3; min–max) used for descriptive statistics and Wilcoxon signed rank test for median comparison. Higher scores on the NRS indicate worse pain. Higher scores on the EQ-5D VAS and RAND-36 indicate better HRQL.

NRS: Numeric Rating Scale for Pain; SD: standard deviation; SED: time spent in sedentary activity; MVPA: time spent in moderate to vigorous activity; HRQL: Health-Related Quality of Life; EQ-5D VAS: EuroQol Five Dimensions visual analogue scale; RAND-36: modified version of Short-Form health survey (SF-36). Patient satisfaction: Satisfaction with pain relief, treatment acceptability, and health care.

*Physical activity.* The median duration of MVPA measured using an accelerometer was 11 min/week (Q1–Q3 = 5.6–39; min–max = 1.8–270) at 9 months and 12 min/week (Q1–Q3 = 5.2–57; min–max = 2.3–263) at 21 months, which did not differ significantly compared with baseline (15, Q1–Q3 = 8.6–49; min–max = 3.6–223) (see Table III). No changes were observed regarding SED during the study follow-ups at 9 and 21 months compared with baseline (*p* = 1.0 and *p* = 1.0, respectively) (Table III). Most participants self-reported engaging in some light PA at baseline (*n* = 17, 61%), and at 9 (*n* = 17, 63%) and 21 months (*n* = 20, 61%) (Table SI). No significant differences were observed in PA outcomes between the follow-ups at 3, 9, and 21 months.

*Health-related quality of life.* HRQL assessed using the EQ-5D VAS showed statistically significant improvements from baseline, from a mean score for those with complete follow-up of 40 (SD = 16) to 56 (SD = 23, *p* = 0.045) at 9 months and 55 (SD = 21, *p* = 0.005) at 21 months (Table III). Scores in the RAND-36 pain domain were improved at 9 (*p* = 0.02) and 21 months (*p* = 0.005) (Table III). Changes in other domains did not remain statistically significant after Bonferroni correction. Fig. 3 displays an overview of the RAND-36 domain scores at different assessment points. There were no statistically significant differences in HRQL outcomes when comparing the 3, 9, and 21-month follow-ups.

**Fig. 3:**
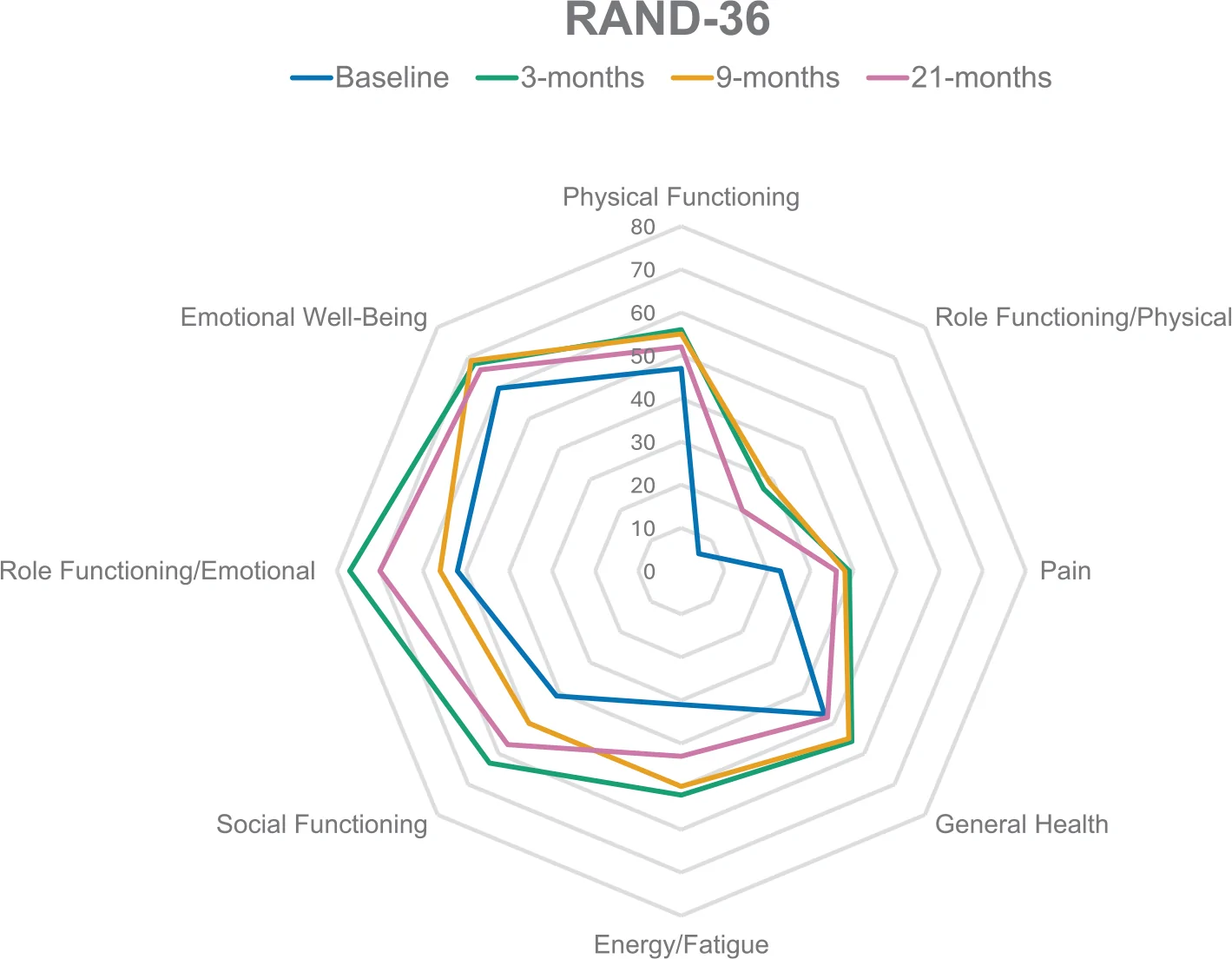
RAND-36 domain scores at different assessment points.

*Additional outcomes.* Patient satisfaction outcomes are presented in Table III. Median patient satisfaction with pain relief was 7.0 (Q1–Q3 = 3.3–8.8; min–max = 0–10) at 9 months and 8.0 (Q1–Q3 = 4.0–8.5; min–max = 0–10) at 21 months. Median treatment acceptability was 7.5 (Q1–Q3 = 4.3–10; min–max = 2–10) and 8.0 (Q1–Q3 = 5.0–9.0; min–max = 0–10) at 9 and 21 months, respectively. Satisfaction with health care was 9.0 (median; Q1–Q3 = 5.3–10; min–max = 2–10) at 9 months and 8.0 (median; Q1–Q3 = 6–10; min–max = 0–10) at 21 months. Self-efficacy and kinesiophobia findings are presented in Table SI; no statistically significant changes were observed for these outcomes.

### Adverse events

All participants who completed the study (*n* = 35) were included in the AE analysis. A total of 84 AEs were reported by 32 participants (Table IV). Of all AEs reported, 31 were deemed directly related to SCS treatment, 10 to the physiotherapist-led exercise programme, 5 to PAP, and 38 to other causes. A total of 27 AEs were judged to be serious; all were CTCAE grade 3 (30). A total of 2 implantable pulse generator (IPG) explants, 1 IPG implant (due to a prior IPG explant previously reported) (19), 4 IPG reoperations, and 2 lead revisions were performed during Phases 2 and 3 of the study.

**Table IV:** Adverse events

Factor	3–9 months after SCS implantation No. of occurrences	9–21 months after SCS implantation No. of occurrences
AE related to SCS treatment		
Technical		
Lead dysfunction	2	
IPG dysfunction		1
IPG displacement	1	2
Biological/Non-technical		
Headache	1	
Other		1
Other complications		
Pain/discomfort over implant	6	3
Pain over incision site	3	
Undesirable stimulation	6	4
Other	1	
Number of AEs related to SCS treatment	20	11
AE related to physiotherapist-led exercise		
Syncope		1
Shoulder pain	4	
Ankle pain	1	
Greater trochanteric pain syndrome	1	
Leg (knee/hip) pain	2	1
Number of AEs related to physiotherapist-led exercise	8	2
AE related to PAP		
Leg (knee/hip) pain		3
Pain over implant after PA		1
Greater trochanteric pain syndrome		1
Number of AEs related to PAP		5
AE due to other causes		
Number of AEs	15	23
Total number of AEs	43	41
Total number of serious AEs (CTCAE grades 3–5)	14	13
Number of patients	26	23

SCS: spinal cord stimulation. AE: adverse event. IPG: permanent implantable pulse generator. PAP: physical activity on prescription. PA: physical activity. CTCAE: Common Terminology Criteria for Adverse Events.

## DISCUSSION

This study found that the improvements in neuropathic pain intensity and HRQL after treatment with SCS remained over time during the period in which participants received the add-on physiotherapist-led exercise programme and subsequent PAP. Our overall findings should be interpreted considering the heterogeneous sample population, with various chronic neuropathic pain conditions, the presence of AEs, and potential progression of comorbidities. Nevertheless, mean changes from baseline in neuropathic pain intensity reached statistical and clinical significance (21, 22), and participants reported increased HRQL, favourable treatment satisfaction and acceptability, despite reports of several AEs. Changes observed in the EQ-5D VAS demonstrated both statistical significance and clinical relevance, with the upper bound of the confidence interval being 2–3 times the MCID (28). Significant long-term improvements were observed in the RAND-36 pain domain, corresponding to increases of 43% (10 points) and 74% (17 points) in the domain score at the 9- and 21-month follow-ups (32). Improvements in the RAND-36 social functioning domain also remained significant over the long term. Further, participants reported a maximal score on the RAND-36 role functioning/emotional domain after 3 months, which was maintained throughout the study period. These changes in HRQL reflect the importance of achieving meaningful pain relief in chronic pain management. The improvement in the social functioning domain of the RAND-36 indicates beneficial effects beyond pain relief and PA measured with accelerometers, which might help explain why patients are satisfied with the treatment despite modest improvements in pain intensity, no detectable changes in PA, and several AEs. This discrepancy prompts consideration of what constitutes a meaningful treatment effect following SCS and highlights the importance of considering multidimensional and patient-relevant outcomes, beyond pain reduction (33).

Notably, no statistically significant differences could be detected when comparing outcomes at the 3-, 9-, or 21-month follow-ups. This indicates that the add-on PA interventions did not augment pain relief or HRQL further, although the improvements after 3 months of SCS treatment were maintained in the long term throughout the duration of the physiotherapist-led exercise programme and PAP. The add-on effects of the PA interventions need to be further assessed in multicentre randomized controlled trials to evaluate their potential contribution to the maintenance of SCS effects. Complementary study designs, such as qualitative studies, are also of importance to capture patients’ goals and to identify meaningful outcomes that capture the beneficial effects of SCS and PA interventions. The findings are consistent with existing evidence on overall neuropathic pain improvements with SCS treatment and recent data regarding the long-term maintenance of clinically important neuropathic pain relief and HRQL after SCS for patients with neuropathic pain of different origins (34, 35). We cannot conclude whether PA interventions contributed to the maintained long-term results, because of the single-arm design and absence of a control group. Our results also showed statistically and clinically significant improvements in pain during movement, addressing the limited data available on this outcome. Lastly, we found no statistically significant differences in objectively or subjectively measured PA levels from baseline at the long-term follow-ups, although we hypothesized that pain relief and improved HRQL would facilitate PA and increase MVPA levels. However, no deterioration in PA levels was observed, although PA levels tend to decrease naturally over time in the general population (36), and the participants’ PA levels were considerably low at all follow-ups (11).

Previous research has relied on subjective self-reported questionnaires evaluating physical functioning, which is often improved following SCS treatment (5). Nonetheless, evidence is mixed on whether objective measures correlate with subjective ones (4, 37). In the present study, we could not detect any objective evidence of improved PA after SCS treatment and the add-on PA interventions. Despite this, physical functioning may improve without corresponding changes in MVPA or PA levels. Notably, Hallén et al. reported increased patient mobility (i.e., upright mobile position ≥ 30 s) 12 months after SCS device implantation for persistent spinal pain after surgery, implying improved daily functional mobility, although they did not directly evaluate activity intensity (4). This outcome was specifically observed in patients with predominant leg pain, which suggests that the underlying pain condition influences the treatment effects on mobility.

In our study, another potential explanation for the observed MVPA results is that participants with neuropathic pain might not pursue increased PA at a moderate or vigorous level, but instead aim to enhance their ability to perform daily activities such as walking, driving, or bending forwards. If these activities were performed more effectively without the interference of pain, consistent with goals and expectations reported by patients before SCS treatment (38), physical functioning and HRQL may have improved without corresponding changes in MVPA levels. Alternately, participants may have compensated for increased PA during the intervention period by reducing other activities and by resting, leaving their MVPA levels unchanged. It is also important to note that high data variability regarding MVPA, including individual PA patterns and levels, may require a larger sample size for detecting statistically significant changes. Our results also align with prior evidence on barriers to changing PA behaviours (39). The physiotherapist-led exercise programme and PAP primarily consisted of structured exercise training but did not specifically address behavioural changes and attitudes towards exercise. It might be beneficial to include interventions that target these issues, as has been discussed in relation to patients with chronic musculoskeletal pain who do not increase PA levels after PA interventions (40). Finally, the residual pain, particularly regarding pain during movement, may have been a barrier for increasing MVPA levels.

In this study, we detected a high incidence of AEs, of which most were of mild severity. This finding is consistent with previous real-world data indicating that SCS has a potentially high burden of AEs, although severe or life-threatening ones occur at a low incidence (41). The most commonly reported AE in the study was pain over the implant, which also aligns with the present literature (41). The high incidence of AEs across all study phases could reflect the clinical nature of our study population and existing comorbidities. AEs are not routinely reported in studies evaluating PA interventions, which limits comparisons across studies. However, reports of AEs related to the add-on PA interventions highlight the importance of providing individualized assistance and follow-up for patients with chronic neuropathic pain, especially those with comorbidities.

The present study is industry-independent and includes a clinical sample of participants with neuropathic pain of different aetiologies treated with SCS, which contributes to the generalizability of the findings. However, the small sample size recruited from a single centre, missing data, and the absence of a control group are important limitations. Caution is warranted in interpreting the results due to the possibility of small study effect on outcomes. Accelerometry captures triaxial acceleration during daily activities and is recommended in research as providing more precise and continuous measurement of bodily movement than self-reported measures. The individualized physiotherapist-led exercise programme and subsequent PAP consisted of PA such as strengthening, balance, core training, cycling, and water-based exercises, which are difficult to capture with accelerometers and may have influenced the results. Furthermore, although individual rehabilitation goals were not systematically addressed, the included PA interventions were individualized based on participants’ preferences and needs to ensure that the intervention was relevant and feasible for participants. Subgroup analysis of responders to PA interventions could not be performed due to the small number of participants achieving ≥ 50% pain relief.

Although this study found clinically and statistically significant beneficial effects on pain and HRQL in the long term, we cannot infer the direct effects of physiotherapy or PAP due to the single-arm design and the absence of a control group. Further studies with larger sample sizes are needed to explore the optimal composition of physiotherapy and rehabilitation interventions, the timing for introducing those interventions in relation to SCS implantation, and appropriate patient selection before recommending routine use of physiotherapy and PAP alongside SCS for chronic neuropathic pain. Patients with neuropathic pain might require extra support and specifically targeted interventions aimed at increasing PA levels and aerobic capacity compared with a general chronic pain population, which could be a target for future research. In this regard, psychological interventions have specifically demonstrated beneficial effects in patients with neuropathic pain and may enhance the observed outcomes of SCS and PA interventions (7). It may also be relevant to further evaluate rehabilitation interventions (e.g., physiotherapist-led exercise and psychological interventions) in combination with SCS, given that SCS is integrated into routine clinical practice for resistant chronic neuropathic pain.

In conclusion, clinically relevant improvements in neuropathic pain intensity, HRQL, and favourable satisfaction after SCS treatment remained at long-term follow-up, with no additional improvements observed beyond 3 months after SCS device implantation. Although an add-on physiotherapist-led exercise programme and PAP were provided during this period, their contribution cannot be determined. PA levels did not change from baseline, indicating that patients with neuropathic pain may require additional support and tailored rehabilitation programmes to increase the intensity and level of PA. Larger studies are likely needed to detect differences in PA levels. Further studies are warranted to evaluate SCS in combination with rehabilitation programmes, comprising not only physiotherapeutic and PA interventions, but also psychological interventions and interventions aimed at behavioural change.

## Supplementary Material



## References

[1] Raja SN, Carr DB, Cohen M, Finnerup NB, Flor H, Gibson S, et al. The revised International Association for the Study of Pain definition of pain: concepts, challenges, and compromises. Pain 2020; 161: 1976–1982. 10.1097/j.pain.0000000000001939. PMC7680716 32694387

[2] Soliman N, Moisset X, Ferraro MC, de Andrade DC, Baron R, Belton J, et al. Pharmacotherapy and non-invasive neuromodulation for neuropathic pain: a systematic review and meta-analysis. Lancet Neurol 2025; 24: 413–428. 10.1016/S1474-4422(25)00068-7. 40252663

[3] Knotkova H, Hamani C, Sivanesan E, Le Beuffe MFE, Moon JY, Cohen SP, et al. Neuromodulation for chronic pain. Lancet 2021; 397: 2111–2124. 10.1016/S0140-6736(21)00794-7. 34062145

[4] Hallén T, Meier K, Kallewaard JW, Billet B, Elzinga L, Schapendonk J, et al. Objective, long-term mobility data in patients with chronic pain after lumbar spine surgery treated with spinal cord stimulation-a prospective, multicenter trial. Reg Anesth Pain Med 2026; 51: 1124–1132. 10.1136/rapm-2025-106726. PMC13638309 40639954

[5] Smet I, Billet B, Germonpré PJ, Peña I, Mendiola de la Osa A, Keiner D, et al. Pain, quality of life, and function in chronic intractable leg pain were substantially improved with 10kHz spinal cord stimulation in a multicentre European study. Eur Spine J 2025; 34: 4900–4912. 10.1007/s00586-025-08752-w. 40192770

[6] Fatima K, Javed SO, Saleem A, Marsia S, Zafar R, Noorani K, et al. Long-term efficacy of spinal cord stimulation for chronic primary neuropathic pain in the contemporary era: a systematic review and meta-analysis. J Neurosurg Sci 2024; 68: 128–139. 10.23736/S0390-5616.23.05930-1. 36943763

[7] Bäckryd E, Ghafouri N, Gerdle B, Dragioti E. Rehabilitation interventions for neuropathic pain: a systematic review and meta-analysis of randomized controlled trials. J Rehabil Med 2024; 56: jrm40188. 10.2340/jrm.v56.40188. PMC11318642 39101676

[8] Vaegter HB, Kinnunen M, Verbrugghe J, Cunningham C, Meeus M, Armijo-Olivo S, et al. Physical activity should be the primary intervention for individuals living with chronic pain: a position paper from the European Pain Federation (EFIC) ‘On the Move’ Task Force. Eur J Pain 2024; 28: 1249–1256. 10.1002/ejp.2278. 38703009

[9] Ghafouri N, Bäckryd E, Dragioti E, Rivano Fischer M, Ringqvist Å, Gerdle B. Effects of interdisciplinary pain rehabilitation programs on neuropathic and non-neuropathic chronic pain conditions: a registry-based cohort study from Swedish Quality Registry for Pain Rehabilitation (SQRP). BMC Musculoskelet Disord 2023; 24: 357. 10.1186/s12891-023-06462-2. PMC10163768 37149571

[10] Caspersen CJ, Powell KE, Christenson GM. Physical activity, exercise, and physical fitness: definitions and distinctions for health-related research. Public Health Rep 1985; 100: 126–131. PMC1424733 3920711

[11] Bull FC, Al-Ansari SS, Biddle S, Borodulin K, Buman MP, Cardon G, et al. World Health Organization 2020 guidelines on physical activity and sedentary behaviour. Br J Sports Med 2020; 54: 1451–1462. 10.1136/bjsports-2020-102955. PMC7719906 33239350

[12] Fjeld MK, Årnes AP, Engdahl B, Morseth B, Hopstock LA, Horsch A, et al. Consistent pattern between physical activity measures and chronic pain levels: the Tromsø Study 2015 to 2016. Pain 2023; 164: 838–847. 10.1097/j.pain.0000000000002773. PMC10026831 36083173

[13] Skogberg O, Karlsson L, Börsbo B, Arendt-Nielsen L, Graven-Nielsen T, Gerdle B, et al. Pain tolerance in chronic pain patients seems to be more associated with physical activity than with depression and anxiety. J Rehabil Med 2022; 54: jrm00286. 10.2340/jrm.v54.241. PMC9131201 35274145

[14] Andersson HI. Increased mortality among individuals with chronic widespread pain relates to lifestyle factors: a prospective population-based study. Disabil Rehabil 2009; 31: 1980–1987. 10.3109/09638280902874154. 19874076

[15] Leitzelar BN, Koltyn KF. Exercise and neuropathic pain: a general overview of preclinical and clinical research. Sports Med Open 2021; 7: 21. 10.1186/s40798-021-00307-9. PMC7984211 33751253

[16] Kallings LV. The Swedish approach on physical activity on prescription. Clin Health Promot 2016; 6 Suppl 2: 31–33.

[17] Joelsson M, Lundqvist S, Larsson MEH. Tailored physical activity on prescription with follow-ups improved motivation and physical activity levels: a qualitative study of a 5-year Swedish primary care intervention. Scand J Prim Health Care 2020; 38: 399–410. 10.1080/02813432.2020.1842965. PMC7782336 33174772

[18] Lundqvist S, Cider Å, Larsson MEH, Hagberg L, Björk MP, Börjesson M. The effects of a 5-year physical activity on prescription (PAP) intervention in patients with metabolic risk factors. PLoS One 2022; 17: e0276868. 10.1371/journal.pone.0276868. PMC9621409 36315564

[19] Gottfridsson R, Varkey E, Wolf A, Gatzinsky K, Liljencrantz J, Thörn SE, et al. Effects of spinal cord stimulation on pain, physical activity, and self-efficacy among patients with neuropathic pain. Pain Manag 2026; 16: 185–199. 10.1080/17581869.2025.2608572. PMC12962678 41503863

[20] Garber CE, Blissmer B, Deschenes MR, Franklin BA, Lamonte MJ, Lee IM, et al. American College of Sports Medicine position stand. Quantity and quality of exercise for developing and maintaining cardiorespiratory, musculoskeletal, and neuromotor fitness in apparently healthy adults: guidance for prescribing exercise. Med Sci Sports Exerc 2011; 43: 1334–1359. 10.1249/MSS.0b013e318213fefb. 21694556

[21] Dworkin RH, Turk DC, Wyrwich KW, Beaton D, Cleeland CS, Farrar JT, et al. Interpreting the clinical importance of treatment outcomes in chronic pain clinical trials: IMMPACT recommendations. J Pain 2008; 9: 105–121. 10.1016/j.jpain.2007.09.005. 18055266

[22] Sabourin S, Tram J, Sheldon BL, Pilitsis JG. Defining minimal clinically important differences in pain and disability outcomes of patients with chronic pain treated with spinal cord stimulation. J Neurosurg Spine 2021; 35: 243–250. 10.3171/2020.11.SPINE201431. 34087802

[23] Arvidsson D, Fridolfsson J, Ekblom-Bak E, Ekblom Ö, Bergström G, Börjesson M. Fundament for a methodological standard to process hip accelerometer data to a measure of physical activity intensity in middle-aged individuals. Scand J Med Sci Sports 2024; 34: e14541. 10.1111/sms.14541. 37985378

[24] Fridolfsson J, Arvidsson D, Ekblom-Bak E, Ekblom Ö, Bergström G, Börjesson M. Accelerometer-measured absolute versus relative physical activity intensity: cross-sectional associations with cardiometabolic health in midlife. BMC Public Health 2023; 23: 2322. 10.1186/s12889-023-17281-4. PMC10668340 37996871

[25] Migueles JH, Cadenas-Sanchez C, Ekelund U, Delisle Nyström C, Mora-Gonzalez J, Löf M, et al. Accelerometer data collection and processing criteria to assess physical activity and other outcomes: a systematic review and practical considerations. Sports Med 2017; 47: 1821–1845. 10.1007/s40279-017-0716-0. PMC6231536 28303543

[26] Grimby G, Börjesson M, Jonsdottir IH, Schnohr P, Thelle DS, Saltin B. The “Saltin–Grimby Physical Activity Level Scale” and its application to health research. Scand J Med Sci Sports 2015; 25 Suppl 4: 119–125. 10.1111/sms.12611. 26589125

[27] Herdman M, Gudex C, Lloyd A, Janssen M, Kind P, Parkin D, et al. Development and preliminary testing of the new five-level version of EQ-5D (EQ-5D-5L). Qual Life Res 2011; 20: 1727–1736. 10.1007/s11136-011-9903-x. PMC3220807 21479777

[28] Shaw C, Longworth L, Bennett B, McEntee-Richardson L, Shaw JW. A scoping review of the use of minimally important difference of EQ-5D utility index and EQ-VAS scores in health technology assessment. Health Qual Life Outcomes 2024; 22: 63. 10.1186/s12955-024-02272-9. PMC11321174 39135171

[29] Shanthanna H, Eldabe S, Provenzano DA, Bouche B, Buchser E, Chadwick R, et al. Evidence-based consensus guidelines on patient selection and trial stimulation for spinal cord stimulation therapy for chronic non-cancer pain. Reg Anesth Pain Med 2023; 48: 273–287. 10.1136/rapm-2022-104097. PMC10370290 37001888

[30] U.S. Department of Health and Human Services. Common Terminology Criteria for Adverse Events (CTCAE), Version 5.0. Washington, DC: U.S. Department of Health and Human Services, National Institutes of Health, National Cancer Institute; 2017.

[31] Ghasemi A, Zahediasl S. Normality tests for statistical analysis: a guide for non-statisticians. Int J Endocrinol Metab 2012; 10: 486–489. 10.5812/ijem.3505. PMC3693611 23843808

[32] Hays RD, Morales LS. The RAND-36 measure of health-related quality of life. Ann Med 2001; 33: 350–357. 10.3109/07853890109002089. 11491194

[33] Goudman L, Billot M, Duarte RV, Eldabe S, Rigoard P, Moens M. Gradation of clinical holistic response as new composite outcome to evaluate success in spinal cord stimulation studies for pain. Neuromodulation 2023; 26: 139–146. 10.1016/j.neurom.2021.10.020.. 35088757

[34] Eldabe S, Nevitt S, Griffiths S, Gulve A, Thomson S, Baranidharan G, et al. Does a screening trial for spinal cord stimulation in patients with chronic pain of neuropathic origin have clinical utility (TRIAL-STIM)? 36-month results from a randomized controlled trial. Neurosurgery 2023; 92: 75–82. 10.1227/neu.0000000000002165. PMC10158909 36226961

[35] Kirketeig T, Söreskog E, Jacobson T, Karlsten R, Zethraeus N, Borgström F. Real-world outcomes in spinal cord stimulation: predictors of reported effect and explantation using a comprehensive registry-based approach. Pain Rep 2023; 8: e1107. 10.1097/PR9.0000000000001107. PMC10653578 38027468

[36] Westerståhl M, Jörnåker G, Jansson E, Aasa U, Ingre M, Pourhamidi K, et al. Rise and fall of physical capacity in a general population: a 47-year longitudinal study. J Cachexia Sarcopenia Muscle 2025; 16: e70134. 10.1002/jcsm.70134. PMC12620399 41243424

[37] Goudman L, Smet I, Mariën P, De Jaeger M, De Groote S, Huysmans E, et al. Is the self-reporting of failed back surgery syndrome patients treated with spinal cord stimulation in line with objective measurements? Neuromodulation 2018; 21: 93–100. 10.1111/ner.12719. 29105225

[38] Goudman L, Bruzzo A, van de Sande J, Moens M. Goal identification before spinal cord stimulation: a qualitative exploration in potential candidates. Pain Pract 2020; 20: 247–254. 10.1111/papr.12845. 31610085

[39] Rhodes RE, de Bruijn GJ. How big is the physical activity intention–behaviour gap? A meta-analysis using the action control framework. Br J Health Psychol 2013; 18: 296–309. 10.1111/bjhp.12032. 23480428

[40] Oliveira CB, Franco MR, Maher CG, Lin CWC, Morelhão PK, Araújo AC, et al. Physical activity interventions for increasing objectively measured physical activity levels in patients with chronic musculoskeletal pain: a systematic review. Arthritis Care Res (Hoboken) 2016; 68: 1832–1842. 10.1002/acr.22919. 27111744

[41] Kirketeig T. Spinal cord stimulation for chronic pain: real-world outcomes and sham-controlled evidence [doctoral thesis]. Uppsala: Acta Universitatis Upsaliensis; 2026.

