# Supplementary material for: LONG-TERM EFFECTS OF SPINAL CORD STIMULATION COMBINED WITH A PHYSIOTHERAPIST-LED EXERCISE PROGRAMME AND PHYSICAL ACTIVITY ON PRESCRIPTION FOR CHRONIC NEUROPATHIC PAIN
Source: J Rehabil Med. 2026 Oct 14;58:46459. doi: 10.2340/jrm.v58.46459 (PMC13673558; doi:10.2340/jrm.v58.46459)
Supplement: Supplementary file 1 [file JRM-58-46459-s1.pdf]

Table SI. Additional secondary outcomes

	Baseline Values All patients (n = 48)	Baseline Values Complete follow-up (n=35)	3 months (n=19-26)	p-value	9 months (n =26-28)	p-value	21 months (n =25-33)	p-value
**Additional secondary outcomes**								
**Physical Activity**								
SGPALS n (%)								
Physically inactive	7 (18)	6 (21)	5 (22)	-	4 (15)	-	6 (18)	-
Some light physical activity	25 (66)	17 (61)	13 (56)	-	17 (63)	-	20 (61)	-
Regular physical activity and training	6 (16)	5 (18)	5 (22)	-	5 (18)	-	7 (21)	-
Regular hard physical training for competitive sports	0 (0)	0 (0)	0 (0)	-	1 (4)	-	0 (0)	-
**Self-efficacy**								
ASES Pain (10-100) median (Q1-Q3; min-max)	49 (34-66; 10-88)	48 (32-67; 10-88)	67 (42-80; 20-100)	0.025*[b]	56 (46-70; 12-100)	1.0[b]	46 (33-62; 10-95)	1.0[b]
ASES Other Symptoms (10-100) median (Q1-Q3; min-max)	55 (39-70; 13-80)	60 (42-70; 13-80)	68 (53-80; 23-93)	0.04*[b]	62 (52-83; 30-100)	0.12[b]	56 (40-68; 10-90)	1.0[b]
SEE (0-90) median (Q1-Q3; min-max)	62 (41-75; 8.0-90)	63 (43-76; 8-90)	71 (47-84; 10-90)	0.85[b]	66 (37-88; 4-90)	1.0[b]	65 (40-84; 7-90)	0.73[b]
**Kinesiophobia**								
TAMPA (17-68) median (Q1-Q3; min-max)	37 (30-43; 23-59)	36 (29-43; 23-52)	32 (28-36; 22-50)	0.9[b]	32 (29-38; 20-56)	0.43[b]	31 (25-41; 20-56)	0.32[b]
TAMPA cut-off > 37 n (%)	19 (51)	13 (48)	6 (24)	-	8 (29)	-	9 (30)	-

Analyses are relative to baseline values. Percentages are based on available data. *p-value significant when < 0.05. p-values are Bonferroni-corrected for multiple comparisons throughout all tests. [a]symmetrical distribution, mean (SD), and t-test for mean comparison. [b]not symmetrical distribution, median (Q1-Q3; min-max) used for descriptive statistics and Wilcoxon signed rank test för median comparison.

SGPALS: Saltin-Grimby Physical Activity Level Scale; ASES: Arthritis Self-Efficacy Scale; SEE: Self-Efficacy for Exercise Scale; TAMPA: The TAMPA Scale of Kinesiophobia. Higher scores on SGPALS, SEE, and ASES indicate higher self-reported physical activity and more self-efficacy. Higher scores on TAMPA indicate more kinesiophobia.
